# An Approach to Derive Functional Peptide Inhibitors of Transcription Factor Activity

**DOI:** 10.1021/jacsau.2c00105

**Published:** 2022-04-06

**Authors:** Andrew Brennan, James T. Leech, Neil M. Kad, Jody M. Mason

**Affiliations:** † Department of Biology & Biochemistry, University of Bath, Bath BA2 7AY, U.K.; ‡ School of Biosciences, University of Kent, Canterbury CT2 7NH, U.K.

**Keywords:** transcription block survival, peptide antagonists, transcription factors, activator protein-1, library screening

## Abstract

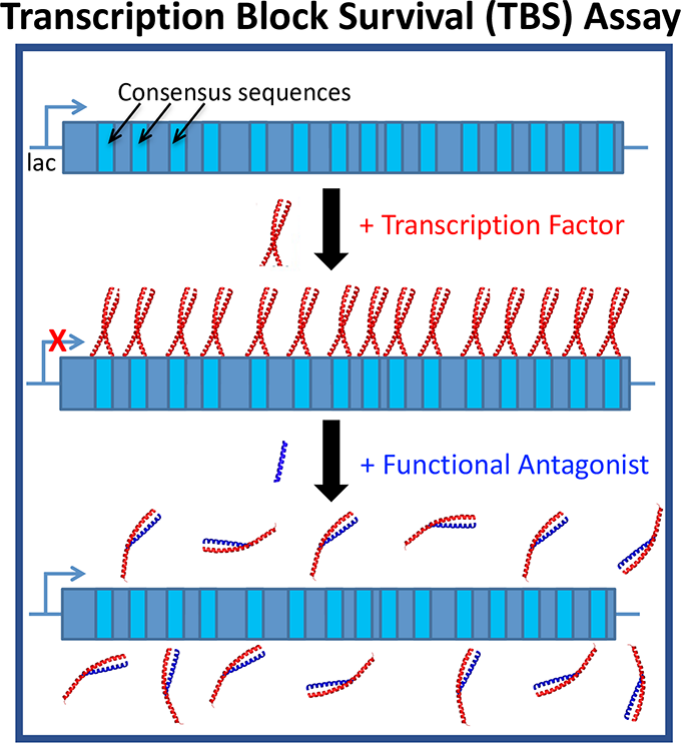

We report the development of a high-throughput, intracellular “transcription block survival” (TBS) screening platform to derive functional transcription factor antagonists. TBS is demonstrated using the oncogenic transcriptional regulator cJun, with the development of antagonists that bind cJun and prevent both dimerization and, more importantly, DNA binding remaining a primary challenge. In TBS, cognate TRE sites are introduced into the coding region of the essential gene, dihydrofolate reductase (DHFR). Introduction of cJun leads to TRE binding, preventing DHFR expression by directly blocking RNA polymerase gene transcription to abrogate cell proliferation. Peptide library screening identified a sequence that both binds cJun and antagonizes function by preventing DNA binding, as demonstrated by restored cell viability and subsequent in vitro hit validation. TBS is an entirely tag-free genotype-to-phenotype approach, selecting desirable attributes such as high solubility, target specificity, and low toxicity within a complex cellular environment. TBS facilitates rapid library screening to accelerate the identification of therapeutically valuable sequences.

## Introduction

**Figure 1:**
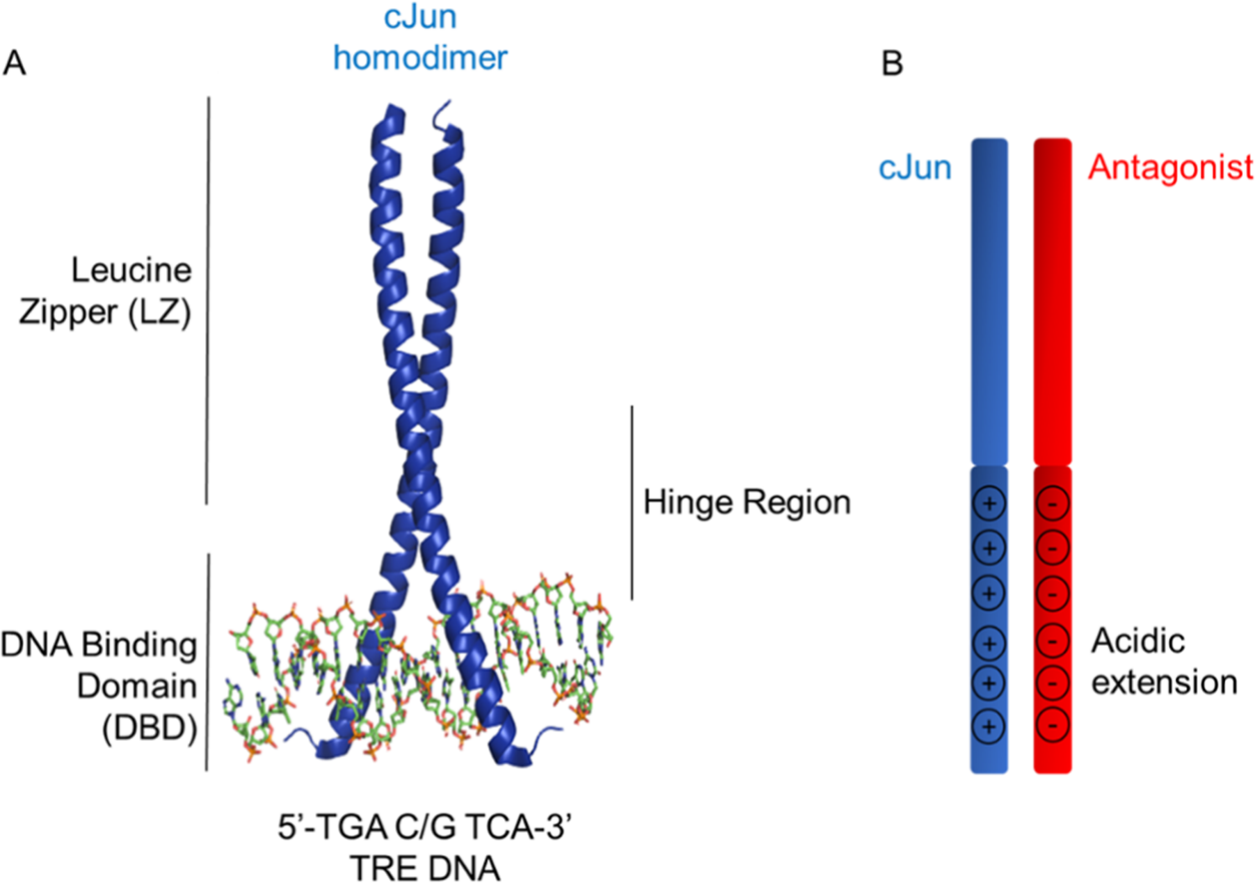
TRE DNA-bound cJun structure and cJun antagonist design. (A) DNA-bound cJun homodimer crystal structure (PDB: 2H7H) is shown to highlight LZ and DBD components required for dimerization and DNA binding. (B) Schematic illustrating the acidic extension design principle (A-FosW, HingeW). This utilizes a region known to bind to the cJun LZ, to which a Glu-rich, extension is appended to interact with the cJun DBD.

Transcription factors (TFs) play crucial roles in the determination of cell function and fate. A range of upstream signals converges upon TFs, converting vital cell signaling processes into transcriptional outputs via specific DNA site recognition. Consequently, of the ∼1600 TFs in the human genome, >300 are associated with a disease phenotype. TF dysfunction leads to a range of detrimental outcomes including cancer, diabetes, and autoimmune and cardiovascular disease.^1,2^ Although there are many upstream points at which TF function can be indirectly modulated, such as via inhibition of kinases or coactivator recruitment, direct and selective TF antagonism is a particularly compelling therapeutic route for the treatment of these diseases, by targeting the end point of dysregulated signaling pathways.^3,4^ TF function is mediated by protein–protein interactions (PPIs) and protein–DNA interactions, which form many points of contact over large surfaces. Small molecules (SMs) have been developed to target relevant DNA sequences, but these interactions are nonselective and have low affinity. Further, SMs typically fail to abrogate these types of interaction since they lack the requisite interaction hotspots, but peptides have the potential to excel as high affinity, selective inhibitors if they can be designed to complement the broad target surface.^5,6^ More than 60% of all multiprotein complexes in the RCSB PDB feature helical PPI interfaces, with at least 20% of those participating in gene regulation. Therefore, helix-based peptide TF inhibitors, in particular, harbor enormous potential for development into a useful class of transcriptional modulators.^7^ In the search for functionally active TF antagonists, we have taken inspiration from the basic leucine-zipper (bZIP) DNA binding mechanism. Dimerization of this domain is driven by the formation of a leucine zipper (LZ), with DNA binding domains (DBDs) extending toward the N-terminus of these helices to facilitate DNA sequence recognition (Figure 1A).^8−11^ Our efforts here focus on developing molecules that inhibit the validated oncogenic transcriptional regulator cJun, a member of the activator protein-1 family and an exemplar for bZIP proteins in general.^12−17^ cJun binds to 12-O-tetradecanoylphorbol-13-acetate response elements (TREs), directly influencing cellular processes such as differentiation, proliferation, and survival.^13−15,18^ Dysregulation of these functions therefore promotes hallmark cancer cell behavior, rendering cJun a focal point for cancer therapy.

Many rational design approaches, library screens, and selection systems exist and have resulted in the successful identification of molecules capable of binding to given TF targets, but a key challenge remains in ensuring that target binding will translate into ablation of function.^17,19^ Selection using a well-studied target exemplar in this work was required to provide suitable antagonists that validate the assay concept. Various methodologies have produced peptide-based cJun antagonists that target the broad LZ binding interface.^20−24^ However, it is difficult to predict if LZ binding will translate into functional antagonism as the cJun DBD remains unbound and capable of binding TRE DNA.^25−27^ A rationally designed peptide has been shown to target the cJun DBD but exhibits lower potency than LZ antagonists, with concerns over specificity due to high sequence similarity across the AP-1 family DBDs.^28^ Similarly, a range of SMs targeting TRE DNA have been developed^29,30^ but these are also lower potency and have the potential to produce off-target effects since multiple TFs typically bind to any given DNA element, with some bZIP/DNA combinations known to promote anti-oncogenic outcomes.^14,31^ One approach to circumvent the potential downsides of these methods is to utilize longer peptides that target the full cJun bZIP domain with a selective yet high-affinity interaction, simultaneously blocking both DNA binding and LZ dimerization. Olive et al. took this approach to produce A-Fos, which combined the wild-type (WT) cFos LZ (known to heterodimerise with cJun) and a rationally designed Glu-rich acidic extension (Figure 1B).^32^ The A-Fos design principle postulated that the LZ interaction is extended N-terminally, generating a DBD-acidic extension interaction facilitated by the incorporation of Leu residues into putative **d** positions in the acidic extension. Here, we develop and validate an intracellular transcription block survival (TBS) library screening assay to search for functional TF antagonists, where cell survival only occurs when TF activity is abolished. Further, bacterial growth rates are correlated with antagonist efficiency allowing for comparison and competition between TF antagonists. We showcase this approach using a peptide library (131,027 members), demonstrating that they can be screened within the TBS platform for functional cJun antagonism. The selected peptide is validated using a range of biophysical approaches indicating a clear improvement from the parent peptide in target binding and cJun/TRE DNA antagonism that is particularly facilitated by a reduction in homodimeric stability.

## Results

### Creation of an Active mDHFR from a TRE Containing Gene to Facilitate a cJun-Imposed Transcriptional Block

**Figure 2:**
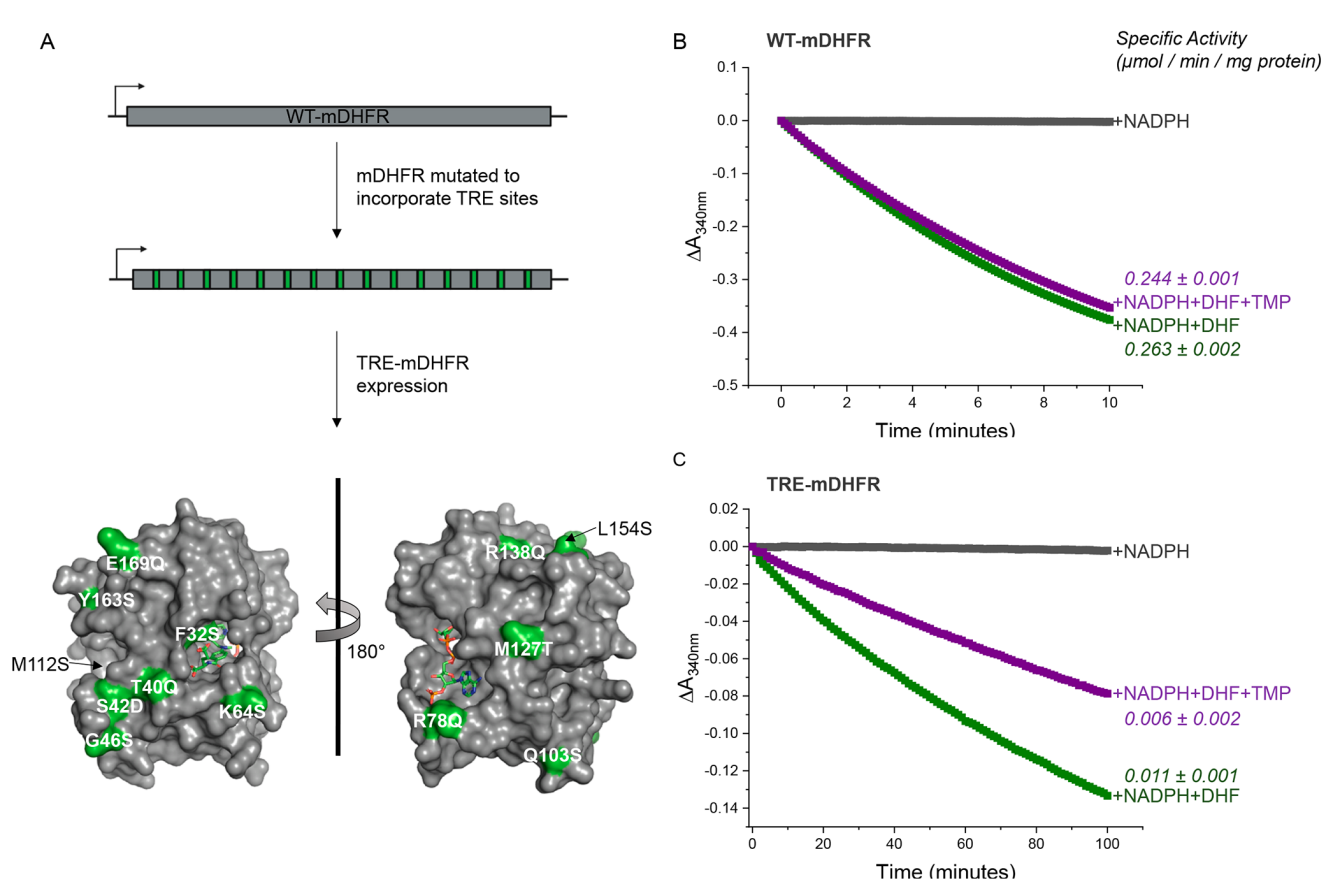
mDHFR retains activity upon introduction of TRE sites. (A) Fifteen TRE sites were introduced into the mDHFR gene (2 silent and 13 substitutions) to allow for a cJun-induced transcriptional block. Substitutions are mapped (green) on the mDHFR structure (PDB code: 1U72) demonstrating surface exposure at positions distal from the active site, where the substrate DHF (shown is competitive inhibitor methotrexate (MTX) bound in the DHF binding site) and cofactor NADPH are bound. Change in absorbance at 340 nm was measured to determine the rate of NADPH turnover by (B) WT-mDHFR and (C) TRE-mDHFR with or without the substrate DHF. Also shown are reactions repeated in the presence of TMP, demonstrating that activity is retained for both enzymes, with TRE-mDHFR partially inhibited as expected. Specific activity was calculated from the linear initial rate (first 2.5 min for WT-mDHFR, first 10 min for TRE-mDHFR; +NADPH only reaction blank subtracted). Data are averages from triplicate experiments with errors shown as one standard deviation. MTX exhibits broader inhibition than TMP, inhibiting both eukaryotic and prokaryotic DHFR enzymes and therefore inhibited both WT- and TRE-mDHFR (Figure S5).

Transcription block survival (TBS) is an intracellular assay that utilizes cell survival as a readout. This allows protein–DNA interaction antagonists to be screened, and the most active identified by their ability to remove a transcriptional block on exogenous murine dihydrofolate reductase (DHFR). This enzyme is absolutely essential for survival since it is required for the production of purines needed for DNA and amino acid synthesis.^33^ Endogenous *Escherichia coli* DHFR (ecDHFR) can be selectively inhibited by trimethoprim (TMP), meaning that cells grown in M9 minimal media are rendered dependent on exogenous murine DHFR (mDHFR) activity for their survival.^34^ We produced an mDHFR gene (Figures 2A and S1) by rational design to introduce 15 TRE sites into the coding DNA sequence to allow a robust cJun transcriptional block while minimizing alteration to the expressed protein (TRE-mDHFR). In particular, the resulting TRE-mDHFR construct was produced via 2 silent and 13 conservative mutations. Using the WT-mDHFR crystal structure as a design guide (PDB code: 1U72),^35^ no changes were made in residues deemed important for 7,8-dihydrofolate (DHF) substrate or nicotinamide adenine dinucleotide phosphate (NADPH) cofactor binding (e.g., A10, L23, W25, and R71^35,36^). The accessible surface areas of all other amino acid residues were calculated using the “Accessible Surface Area and Accessibility Calculation for Protein*”* tool.^37,38^ A cutoff score of 20 Å^2^ was implemented, below which residues were deemed to be buried from solvent exposure and therefore more likely to cause disruption if changed. Of the remaining nonessential, surface-exposed residues, mutations were only permissible where the R group change was relatively conservative. The precise permitted substitutions included to incorporate TRE sites were as follows: F32S, T40Q, S42D, G46S, K64S, R78Q, Q103S, M112S, N127T, R138Q, L154S, Y163S, and E169Q.

### Establishing a Transcription Block Survival Assay

We first sought to confirm whether the new TRE-mDHFR construct could replace the TMP-inhibited ecDHFR by confirming it expresses, folds, and is catalytically active. This was achieved via (i) SDS-PAGE analysis of cell lysate, confirming that the protein is expressed within the soluble fraction upon isopropyl β-D-1-thiogalactopyranoside (IPTG) induction (Figure S2), (ii) plating *E. coli* containing the TRE-mDHFR plasmid onto M9 agar supplemented with TMP; no growth was observed (4 μM TMP, optimized in Figure S3), with growth restored upon induction of TRE-mDHFR expression by IPTG (Figures S4 and 3B-3), and (iii) purified recombinant WT- and TRE-mDHFR activity was monitored by following the reduction of NADPH at 340 nm in the presence of the DHF substrate (Figure 2B,C). The specific activities calculated from these reactions demonstrated a 24-fold reduction in activity for TRE-mDHFR relative to WT. In addition, TRE-mDHFR showed a ∼1.8-fold reduction in specific activity in the presence of TMP, whereas WT-mDHFR was unaffected. Despite an expected reduction in activity resulting from the 13 amino acid substitutions, TRE-mDHFR retained its ability to turnover DHF and impart survival (while ecDHFR is compromised), confirming its suitability for TBS.

**Figure 3:**
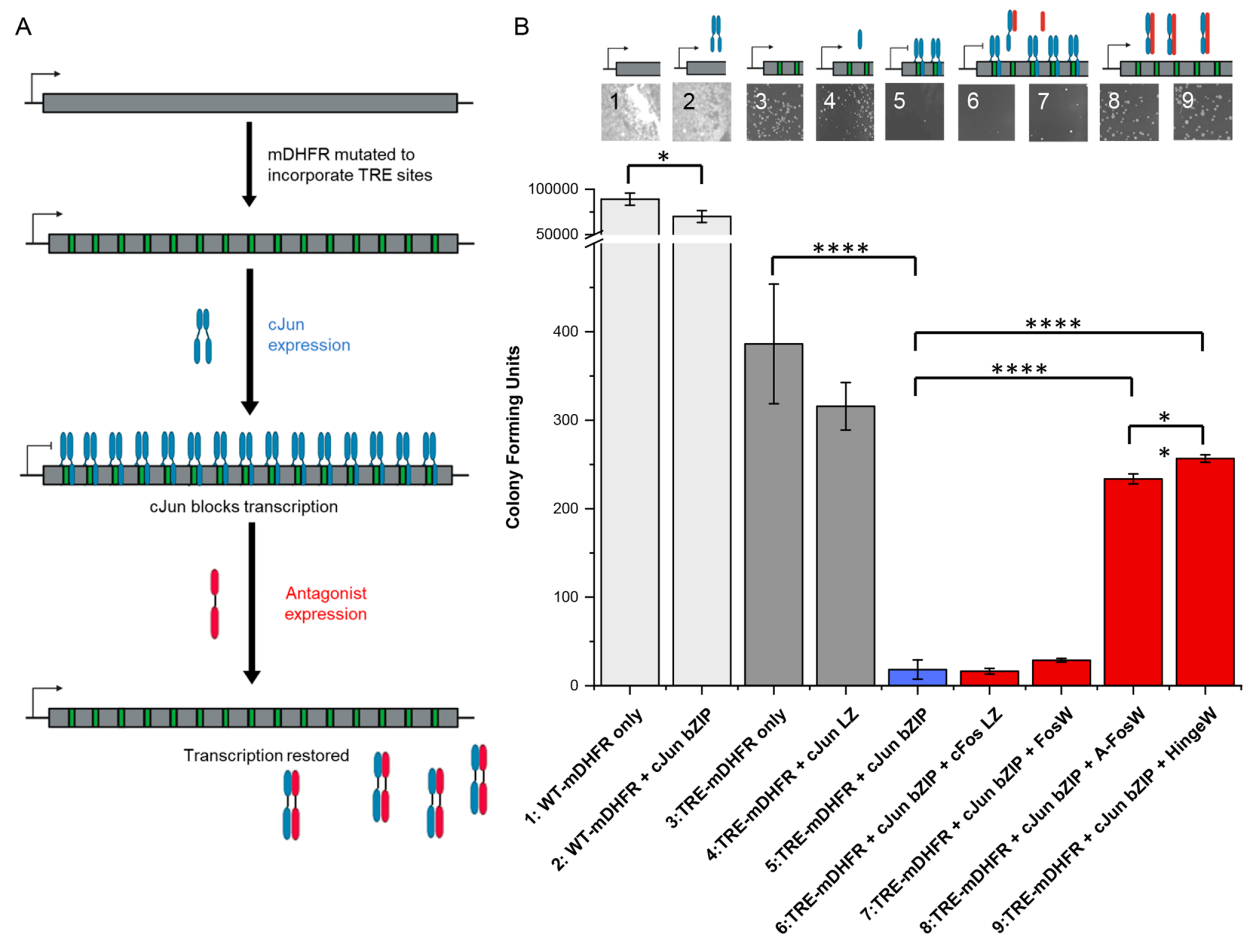
Transcription block survival (TBS) assay to derive functionally active cJun inhibitors. (A) Schematic illustrating the design and operation of TBS. (B) Controlled numbers of *E. coli* expressing the indicated proteins were plated on selective media and growth rates were calculated by counting colony-forming units. (1) WT-mDHFR expression can replace ecDHFR and is uninhibited by TMP producing significant growth. (2) A small effect on colony numbers is observed when cJun bZIP is additionally expressed. (3) TRE-mDHFR can replace the inhibited ecDHFR with colony count lower than for WT as expected. (4) The cJun LZ domain (lacking DBD) does not affect TRE-mDHFR transcription and colony formation; however, (5) the cJun bZIP domain (with DBD) binds TRE sites to block transcription of TRE-mDHFR, leading to reduced bacterial survival. Although (6) cFos LZ and (7) FosW are known cJun-binders, they are unable to effectively dissociate the cJun bZIP from TRE DNA. However, (8) A-FosW and the TBS-derived hit (9) HingeW remove TRE-mDHFR transcriptional blocks to restore cell survival. Bar charts represent averages of three experimental repeats. Errors are shown as one standard deviation. Selected *P* values from a t-test are indicated (*P ≤ 0.05; **P ≤ 0.01; ****P ≤ 0.0001) with values for all possible comparisons within the bar chart reported in Figure S7. Serial dilutions were used to quantify colony numbers where required. Also shown are representative plate images and schematics to illustrate the effect upon TRE-mDHFR transcription.

**Figure 4:**
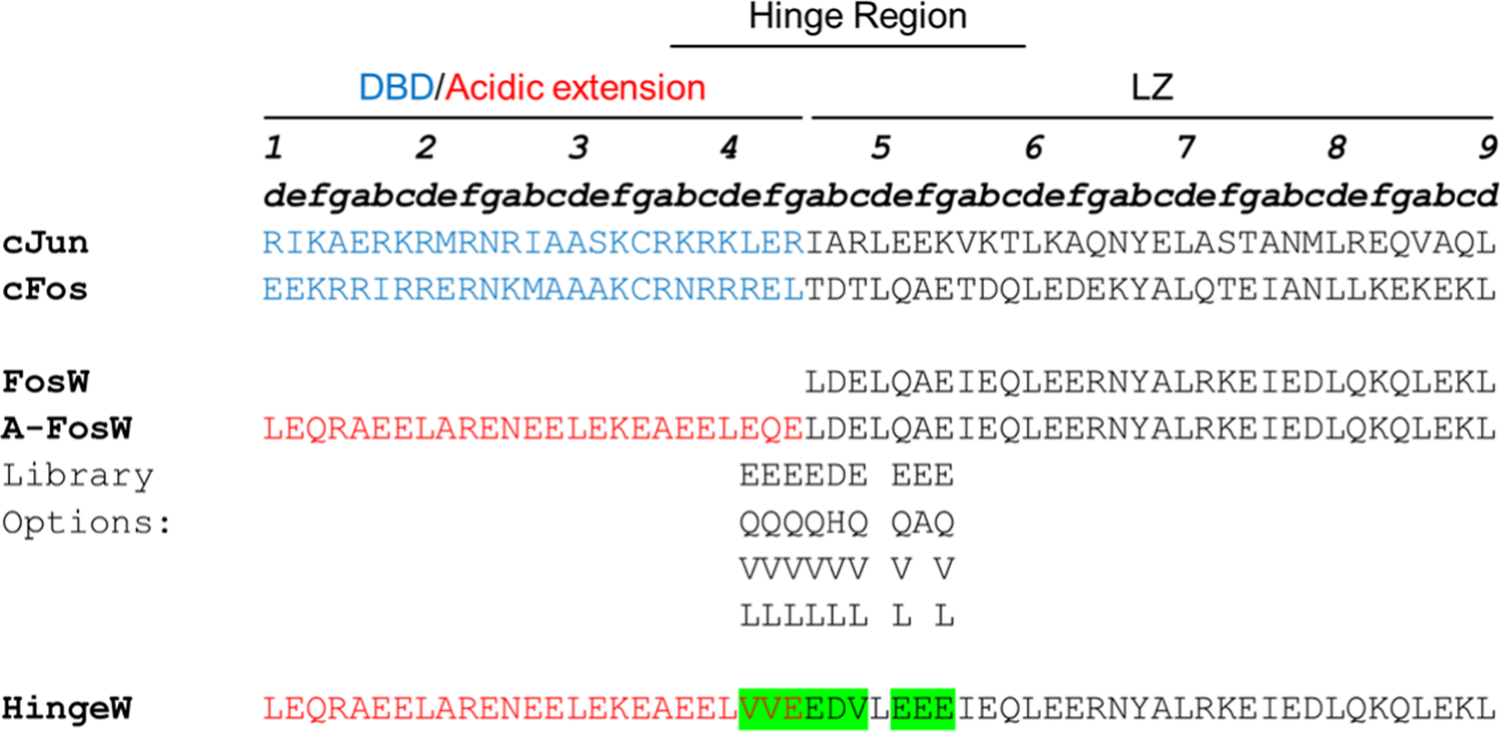
Target and antagonist peptide sequences and TBS library design. The cJun target sequence is shown and compared to related off-target cFos. To facilitate optimization of cJun binding, nine residues within a 10-residue tract (**e4**–**g5**) in the A-FosW sequence were selected for variation within the library, providing acidic, polar, and hydrophobic options, resulting in a 131,072-member library. Screening using TBS produced the “HingeW” sequence. DBD and acidic extension regions are shown in blue or red, respectively, with the selected library options in the winner peptide highlighted in green. Residues are named according to the heptad numbering and position within a given heptad repeat.

**Figure 5:**
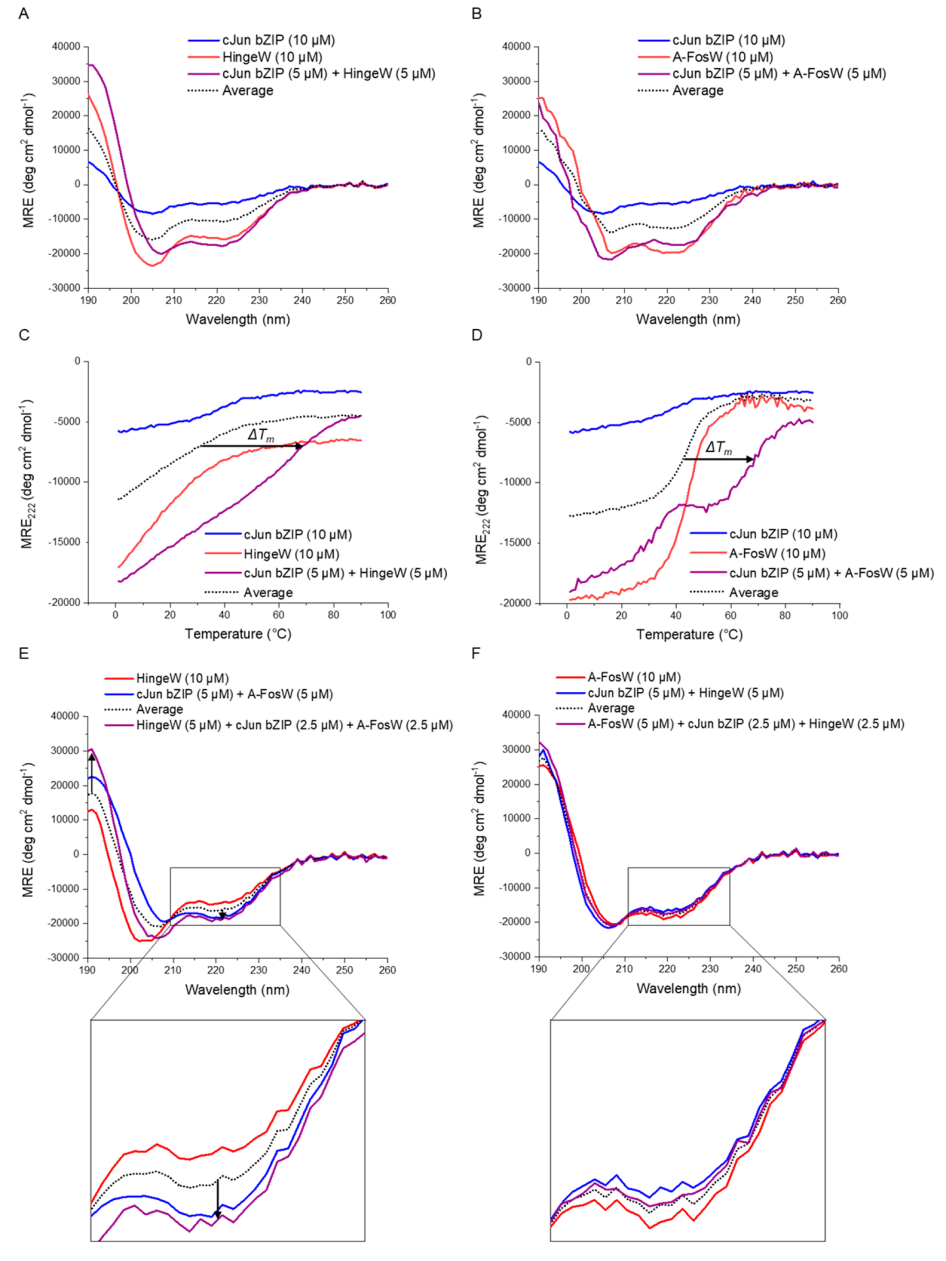
TBS winner peptide HingeW binds cJun preferentially over A-FosW. CD spectra (20 °C) show the binding of cJun to either (A) HingeW or (B) A-FosW. In both cases, the heterodimeric spectrum shows increased α-helical character relative to the average of the component peptides (the expected spectrum for no interaction). However, the effect is larger for HingeW, indicating a greater increase in peptide helicity. Similarly, the thermal denaturation of cJun bound to either (C) HingeW or (D) A-FosW is right-shifted from the average of the component peptide denaturation profiles. HingeW/cJun displays a larger Δ*T*_m_ of binding than A-FosW/cJun due to the lower *T*_m_ of the HingeW homodimer (indicated by arrows). CD dimer exchange spectra show (E) an increase in helicity when HingeW is mixed with the A-FosW/cJun heterodimer as the HingeW exchanges with the A-FosW due to the binding preference of cJun for HingeW and (F) no shift from the average is observed when A-FosW is mixed with the HingeW/cJun heterodimer, indicating no change in dimer populations. Arrows are shown to highlight the shift from the average at 190 and 222 nm. In all experiments, the total sample peptide concentration was fixed to 10 μM, using equimolar concentrations of each component peptide to remove concentration-dependent effects. Data is summarized in Table S3.

Having established that TRE-mDHFR is active and absolutely required for cell survival under selective conditions (M9 minimal media +4 μM Tmp + 1 mM IPTG), we next expressed the cJun bZIP domain in cells containing the TRE-mDHFR plasmid, which resulted in a 21-fold reduction in colony counts (*P* ≤ 0.0001; Figure 3B-5). Expression of cJun bZIP in the presence of WT-mDHFR (i.e., lacking the requisite TRE binding sites) reduced bacterial proliferation (*P* ≤ 0.05, Figure 3B-1 vs 3B-2). This is presumably due to overexpressed cJun binding nonspecifically to the plasmid DNA. However, the transcription block is strongly TRE site-specific, as indicated by the small 1.3-fold reduction without TRE sites. As a further control, we also introduced a cJun LZ only construct, in which the 25 residue DBD was deleted. This peptide was unable to initiate DNA binding and, as expected, did not affect bacterial colony formation (*P* = 0.1, Figure 3B-4 vs 3B-3). Taken together, this specifically correlates the interaction between the cJun bZIP and TRE sites with ablation of bacterial growth within the TBS system, validating that any subsequent increase in bacterial growth is due to inhibition of this interaction.

**Figure 6:**
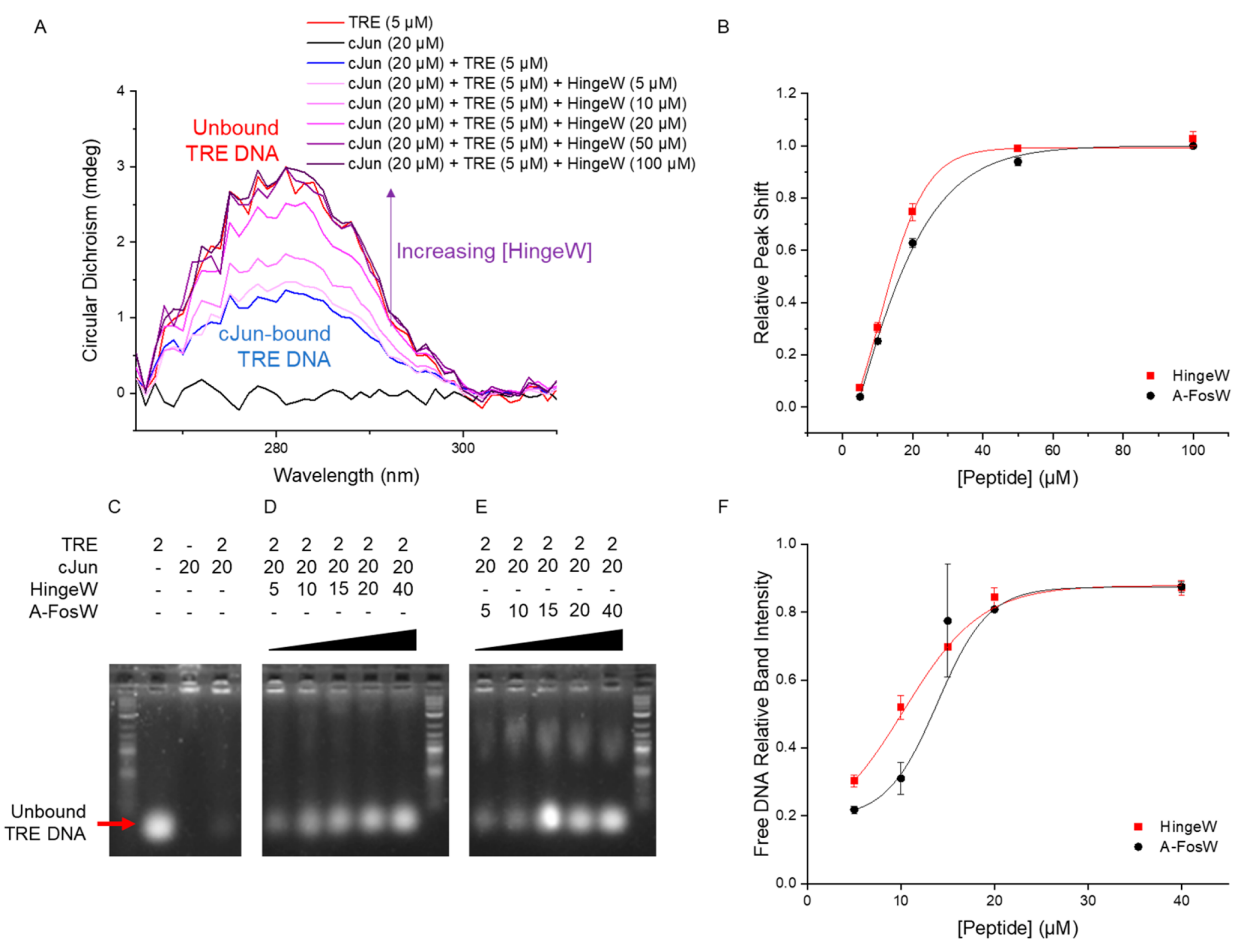
HingeW antagonizes cJun/TRE DNA interaction more effectively than A-FosW. (A) CD spectra showing a shift in the TRE DNA peak at ∼281 nm upon addition of cJun, which is reversed by the titration of HingeW into the sample, as HingeW sequesters the cJun in a nonfunctional heterodimer. (B) Relative peak shift from bound to free TRE is plotted for varying concentrations of HingeW and A-FosW, showing greater cJun/TRE DNA inhibition for HingeW across all concentrations. EMSA showing the (C) unbound TRE DNA band shift upon addition of cJun and the subsequent restoration of the unbound DNA band intensity upon titration of either (D) HingeW or (E) A-FosW. (F). For both CD and EMSA, data was averaged from three independent experiments and the plotted error bars indicate one standard deviation.

Next, peptides known to bind to cJun were introduced into the system, to establish whether they can impact cJun function—i.e., sequester the cJun bZIP as a nonfunctional heterodimer, therefore preventing DNA binding and rescuing TRE-mDHFR transcription. Here, we used two peptides targeting the cJun LZ domain: cFos LZ and FosW, an optimized sequence identified from a protein-fragment complementation assay (PCA) that readily binds to cJun in the absence of DNA at nM affinity.^21,39^ Despite their known interactions with cJun, both peptides were shown to be ineffective in restoring TRE-mDHFR expression and activity, producing no significant increase in colony numbers from the transcriptionally blocked cells (*P* > 0.05 in both cases, Figures 3B-6 or 3B-7 vs 3B-5). This important finding demonstrates that although FosW can outcompete the cJun dimer to form a nonfunctional heterodimer, it is unable to free DNA-bound cJun from TRE sites within TRE-mDHFR.

To address this, we turned to work by Olive et al., in which antagonism of cJun was achieved using Acidic-cFos (A-Fos), whereby a rationally designed acidic extension was appended to the cFos LZ.^32^ Since FosW was shown to improve binding to the cJun LZ relative to the WT cFos LZ sequence in the absence of DNA,^21^ an improved hybrid construct was rationally designed. This blended the two previously published components by appending the rationally designed acidic extension to the N-terminus of the FosW LZ sequence to generate A-FosW (Figure 4). This protein was designed to act as a template for peptide library design and optimization using TBS screening. Reassuringly, the template peptide was able to successfully antagonize the cJun/TRE DNA interaction, restoring 60% of the colony numbers relative to TRE-mDHFR only (Figure 3B-8 vs 3B-5). Importantly, all experimental variations above were plasmid-matched with appropriate dummy constructs to control for potential differences in antibiotic stress (Table S1). TBS assay design is summarized in Figure 3A.

### Hinge Library Design

The acidic extension design principle is the most successful methodology in the literature to target the full bZIP domain of various proteins.^32,40,41^ However, incomplete restoration of colonies using A-FosW indicated that transcription remained partially hampered by cJun binding across the 15 TRE sites. This allowed us to employ TBS to screen a peptide library, using A-FosW as a design template, toward further improvement in cJun/TRE DNA antagonism. The library design utilized semirandomized positions within the hinge region that straddles the acidic extension and LZ domains (Figure 4). It was anticipated that optimization in the hinge region would induce a significant increase in functional antagonism with high-affinity binding simultaneously disrupting cJun LZ dimerization and, by scrambling at the area between the two domains, providing a more effective block of the DBD–DNA interaction. Previous studies have generally sought to inhibit binding at either the DBD or LZ, but here we aim to target both regions to generate an improved antagonist versus targeting either domain separately. Of the nine semirandomized hinge positions, four options were included across eight positions (**e4**, **f4**, **g4**, **a4**, **b4**, **c4**, **e5**, **f5**, and **g5**): two hydrophobic (V/L), one acidic (E or D) and one polar (Q or H). Options of A/E were included at **f5** as an **f** position in the LZ region since these positions were deemed unlikely to be involved in direct target interaction; thus, these residues were presented to assist with solubility or higher helical propensity to enhance the PPI by entropic preorganization. Experimental limitations prevent more amino acid diversity from being incorporated at so many positions; however, these selections will allow testing for general amino acid preference. In all positions, parental residues of A-Fos were incorporated into the library so that this functionally active benchmark protein was included within the screen. Restriction from the genetic methodology used to produce the library result in different mixtures of residues at different positions. Leu at **d5**, in the middle of this stretch of semirandomized positions, remained unchanged since alteration was deemed unlikely to be favorable or may favor other coiled coil architectures than the desired parallel dimer. Altogether, this produced a peptide library of 131,072 members (Figure 4).

### TBS Library Screening

During the TBS library screening process, *E. coli* were transformed with the pooled DNA plasmid library such that each cell expressed a given member. Cells were plated onto M9 selective media and incubated until colonies expressing TBS active library members had formed. These colonies were pooled, and repeated liquid culture passages were undertaken under selective conditions to compete for library members against each other, enriching for the most TBS active sequences. At each stage of the assay, DNA sequencing was used to monitor the presence of TBS active sequences in the culture and inform on which residues were being selected at each position until one discrete DNA sequence was detected in the culture, referred to as HingeW (Figure 4). The residues selected in the winning HingeW peptide were universally found to be either acidic (D/E) or hydrophobic (V). Six of nine residues selected deviated from the parental A-FosW sequence with both V (E-to-V at **e4** and **c4**, Q-to-V at **f4**) and E (L-to-E at **a4**, Q-to-E at **e5**, A-to-E at **f5**) newly selected in three positions. The shift in the proportion of library options at each position during competition rounds provides information on how favored a particular residue or residue type may be as the selection progresses (Figure S6). Two residues were selected after the first passage and were therefore considered particularly important for optimization of this interaction: V at **f4 (**Q in the parent sequence) and E at **g4** (unchanged). At position **f5**, there was little preference for either option with E selected over A (A in the parent sequence) at the end of the second passage, indicating less impact on target binding. Although the sequence for A-FosW was included within the library, further confirmation of the TBS selection preference for HingeW over the parental sequence was undertaken by direct competition in liquid culture. In this experiment, equal numbers of TBS cells containing either A-FosW or HingeW were mixed in selective M9 media and subjected to competition selection. After three passages, only HingeW was observed via DNA sequencing, as the A-FosW-containing cells had been outcompeted. This was further supported in TBS colony counting experiments, which showed a 10% increase in colony numbers for HingeW relative to A-FosW (*P* = 0.009, Figure 3B-9 vs 3B-8), rising to 66% of the theoretical maximum colony numbers observed for TRE-mDHFR alone (Figure 3B-3).

### HingeW Binds cJun Preferentially over A-FosW

Experiments were next undertaken to compare the binding of A-FosW and TBS-optimized HingeW to the cJun bZIP. CD spectroscopy was utilized to measure the global secondary structure of homo- and heterodimeric peptide samples, providing information on global α-helicity and thermal denaturation temperatures (*T*_m_). The HingeW/cJun spectrum was 82% higher in α-helical content relative to the average of the two-component spectra (Figure 5A). The average is the predicted spectrum for no interaction between the two components. This occurs as the total peptide concentration of the sample is kept constant (10 μM), meaning that the concentration of each component is halved upon mixing. If a sample component structure is unchanged upon mixing (i.e., no binding occurs), the CD signal for each component will average. The same, though smaller, trend was observed from the A-FosW/cJun spectra, where the α-helicity was 39% greater than the average (Figure 5B). This increased α-helical gain upon binding of HingeW/cJun implies a higher affinity interaction. Of note is that HingeW in isolation is 12.9% less helical relative to A-FosW. The AGADIR helical propensity calculator was used to calculate predicted helicity scores of 14.5 and 13.2 for A-FosW and HingeW, respectively.^42^ The observed difference in heterodimeric peptide helicity may be partially explained by A-FosW being inherently more helical, but the larger scale of the observed effect than this prediction can likely be explained by a homodimeric preference for A-FosW relative to HingeW.

Thermal denaturation analysis of HingeW/cJun, following the loss of signal at 222 nm, displayed a *T*_m_ of 71.2 °C for the HingeW/cJun heterodimer, representing a clear increase from the *T*_m_ of the two-component denaturation profiles (Figure 5C). For A-FosW/cJun, the *T*_m_ was observed as 69.9 °C (Figure 5D). Although this represented a negligible 1.3 °C increase in heterodimer *T*_m_, there was crucially a much larger Δ*T*_m_ for HingeW/cJun relative to the component peptide denaturation profiles than for A-FosW/cJun. The low thermal stability of the HingeW homodimer results in no observable lower baseline prior to the transition such that the *T*_m_ for this component, and thus the average, cannot be determined. However, this Δ*T*_m_ can be estimated to be ∼40 °C, compared to 27.5 °C for A-FosW/cJun. The TBS screen has therefore led to an optimized reduction in homodimerization more so than increased heterodimerization with the target. This ensures that more antagonist is available as free monomer in solution and therefore in a target-dimerization competent state. Another difference between the two denaturation profiles is the presence of a double transition for the A-FosW/cJun heterodimer, with a smaller initial transition occurring at ∼30 °C. Jain et al. have previously reported a double transition in similar acidic extension antagonist/bZIP denaturation profiles and suggest that the lower temperature transition occurs due to fraying of the N-terminal acidic extension/DBD interaction, with the higher temperature transition corresponding to dissociation of LZ regions.^43^ Crucially, these two novel antagonists have significantly higher target heterodimer *T*_m_ values than FosW (*T*_m_ = 54 °C, Figure S7). This demonstrates a clear benefit from the inclusion of the acidic extension, which is absent in FosW. Importantly, due to sequence differences between cJun and cFos (Figure 4), optimization for cJun binding means that HingeW displays no interaction with the cFos bZIP domain (Figure S8).

### HingeW Outcompetes A-FosW for cJun Binding

Direct competition between HingeW and A-FosW for cJun binding was observed using CD dimer exchange experiments in which a solution containing one antagonist/cJun mixture was combined with the other antagonist to observe potential changes in α-helicity, as an indicator of a change in cJun dimerization partner. In this case, when HingeW was mixed with the preformed A-FosW/cJun heterodimer, a 17% increase in helicity was observed, as measured at 222 nm, relative to the average of the component signals (Figure 5E). There was also a clear increase in the 190 nm peak relative to the average of the two-component peptide spectra. This change is significant and indicates a clear change in structure, and therefore a dimer exchange, whereby the cJun which was bound to A-FosW, is now bound to HingeW. Reversing the experiment and mixing A-FosW with a preformed HingeW/cJun heterodimer produced a measured spectrum that overlaid with the average of the components, indicating that no dimer exchange had occurred (Figure 5F). In combination with TBS growth competition data, the dimer exchange experiments strongly suggest preferential binding of cJun to HingeW relative to A-FosW when in competition.

### Isothermal Titration Calorimetry Demonstrates Improved Binding Affinity for HingeW

The binding interactions of cJun with HingeW and A-FosW were further studied by ITC, to provide information on the thermodynamic parameters (Figure S9). The data produced from the injection of HingeW into cJun were fit to a single-site binding model (*N* = 1.05 ± 0.05) with a *K*_D_ of 14.4 ± 3.7 nM and a Δ*H* of −85.4 ± 4.5 kJ mol^–1^ (*T*Δ*S* = −39.2 ± 4.5 kJ mol^–1^). A-FosW binding to cJun was also fit to a single-site model (*N* = 1.04 ± 0.08) with a *K*_D_ of 88.3 ± 17.6 nM and a Δ*H* of −152.6 ± 4.1 kJ mol^–1^ (*T*Δ*S* = −112.6 ± 4.1 kJ mol^–1^). This confirms the predicted 1:1 binding stoichiometry of both interactions while demonstrating a 6-fold increase in binding affinity upon TBS optimization of A-FosW to HingeW. Both interactions are enthalpically driven with negative entropic contributions. The entropic component is significantly more unfavorable for the A-FosW interaction, which may indicate some entropic preorganization for HingeW.

### HingeW Effectively Antagonizes the cJun/TRE DNA Interaction

The binding of cJun to TRE DNA can be observed by monitoring a DNA absorbance peak in the CD spectrum centered at ∼281 nm.^44^ Peptides (cJun, HingeW, or A-FosW) in isolation do not absorb at this wavelength, meaning that all changes in the spectrum in this region correspond to shifts in DNA conformation. The addition of cJun (20 μM) to TRE DNA (5 μM) decreases this DNA peak by 55% as the cJun engages its target TRE site and alters the DNA structure (Figure 6A). Subsequent titration of HingeW into this bound cJun/TRE DNA mixture reverses the peak shift, with the peak increasing as DNA is released. This occurs in a dose-dependent manner until the signal overlays with the free DNA spectrum at HingeW concentrations of 50 and 100 μM, indicating complete antagonism of the cJun/TRE interaction. Plotting and fitting the relative peak shifts to the Hill equation (OriginPro) yields an IC_50_ of 13.4 ± 0.6 μM, which can be compared to the equivalent data for A-FosW antagonism, which produces an IC_50_ of 16.0 ± 0.4 μM (Figure 6B). This shows significant improvement in both cases over FosW, which lacks an acidic extension, and displays an IC_50_ of 119.8 ± 1.1 μM (Figure S10). In control experiments, both HingeW and A-FosW were shown to have no interaction with DNA (Figure S11).

To provide further evidence of functional antagonism, an electrophoretic mobility shift assay (EMSA) was employed. First, cJun bZIP (20 μM) was mixed with the TRE DNA construct (2 μM), resulting in a significant reduction in the free DNA band intensity relative to DNA alone (Figure 6C). No bound cJun/TRE DNA band was observed as the overall charge of this complex prohibited entry into the gel. Antagonism was therefore best observed by monitoring the intensity of the free DNA band. A concentration-dependent increase in the free DNA band intensity was observed upon the addition of HingeW to cJun/TRE DNA (Figure 6D). The same trend was observed for increasing concentrations of A-FosW with cJun/TRE DNA (Figure 6E). In close agreement with the CD DNA peak analysis, the data could be fit to the Hill equation (OriginPro) to determine an IC_50_ value of 9.6 ± 0.8 μM for HingeW and 12.1 ± 1.9 μM for A-FosW (Figure 6F).

## Discussion

There are many screening platforms in place to derive high-affinity PPIs, but none that guarantee target binding will lead to the desired loss-of-function of the target protein. Using cJun/TRE as an exemplar, we have developed a transcription block survival assay that has the potential to be used as a generalized approach for the derivation of peptides capable of ablating TF activity. We have engineered a “molecular dial” into a bacterial system, whereby the cJun/TRE DNA interaction is inversely correlated with cell proliferation. By introducing cJun/TRE antagonists into this system, cellular growth becomes a direct readout for the ability of the antagonist to functionally block the numerous cJun/TRE interactions, turning the molecular dial up. The most effective rationally designed acidic antagonist was next utilized as a parental sequence to design a semirandomized library that was successfully screened in the TBS platform, to produce the in vitro validated assay hit HingeW.

Establishing the TBS system required the production of a mutant DHFR gene (TRE-mDHFR), which retained its enzymatic activity upon introduction of 15 TRE sites into its DNA sequence, leading to 13 amino acid substitutions. This allowed for a cJun-induced transcriptional block when the TF binds to the TRE sites on the TRE-mDHFR plasmid DNA. For loss of TRE-mDHFR activity to take place, there is an absolute requirement for both the TF DBD and the TRE sites within the mDHFR gene, confirming specificity in the TBS system. The phenotype of bacterial growth rate is directly linked to the genotype of the antagonist sequence expressed by virtue of the system’s containment within a single cell. Bacterial cells are ideal for this process owing to their fast growth rate, durability, ease of use, and low cost. Crucially, they also allow for the direct measurement of cJun interacting with TRE sites in the absence of any related eukaryotic TFs that might interfere with the assay. More work is needed to verify that TBS assay hits can translate to mammalian systems; however, the major barrier is defining an inhibitor. Further modifications aimed at making these peptides compatible with mammalian systems will be the next step of inhibitor development, although the in-cell mode of selection used here should favor this. Furthermore, many in vitro screening systems have been widely adopted in the drug development pipeline, as evidenced by the widespread use of phage display methodologies and related mRNA and ribosome display screening systems.

TBS facilitates high-throughput genotype-to-phenotype screening and competition of peptide libraries to isolate those that result in functional loss of cJun DNA binding activity from those that bind but have little or no effect upon target activity (or those that do not bind at all). This distinction is important since it means that an antagonist must not only bind to the target free in solution but must also be capable of meeting the much more demanding task of liberating the TF from DNA, which is known to be more stable.^45^ Lastly, all of the above is undertaken within the complex environment of the cytoplasm, removing molecules that are toxic, nonspecific, insoluble, or protease susceptible from consideration at the initial screening stage, rather than determining this at later hit validation or clinical trial stages. These factors are particularly important for longer peptides, such as those required to bind to the large and shallow cJun bZIP surface, which tend to lack these important qualities. TBS improves upon the related protein-fragment complementation assay, as well as in vitro screening platforms such as phage display or ribosome display, by the complete removal of any requirement for bulky protein fusions or hydrophobic/aromatic tags, which can interfere with the relevant assay interactions and lead to false readouts.

The central advantage of TBS is the requirement for assay hits to prevent TFs from binding to their consensus DNA sequence as exemplified by the combined design of A-FosW, a hybrid containing domains from both A-Fos^32^ and the FosW PCA hit.^21^ In A-FosW, the LZ targets the antagonist to the cJun bZIP with high affinity and selectivity, with the acidic extension added to assist in functionally antagonizing the cJun/TRE DNA interaction by blocking the cJun DBD. The LZ domains of bZIP proteins tend to display more sequence diversity than the DBD, which is useful for therapeutic targeting of specific AP-1 family members, providing better control and potentially fewer side effects.^14^ Although it is unclear if A-FosW binds cJun by forming a single continuous LZ interaction as designed, increased binding around the hinge region of cJun was anticipated to propagate increased helicity and therefore affinity in either direction. Further, focusing on the hinge region was supported in the original work of Olive et al., where a point mutation in this region of A-Fos (N26L at position **a4** of A-FosW) produced a significant increase in cJun binding affinity and subsequent cJun/TRE antagonism.^32^ Optimization of the acidic extension through rational design is hampered by the lack of design rules for guidance, as is the case for the LZ domain, which has known structure and predicative tools to produce high-affinity interactions.^20,22^ Additionally, no library-based approach had previously been used to optimize binding within this region of cJun. Using A-FosW as a design template and including library options in the hinge region was a clear next step which resulted in the TBS selection of HingeW, with 14 nM affinity for the target cJun protein (a 6-fold improvement over A-FosW). HingeW included one more acidic residue than A-FosW, supporting the Olive et al. methodology^32^ of including dominant negative charge throughout the N-terminal domain to interact favorably with positive charge within the cJun DBD. However, the precise selection pattern was more nuanced than simply producing a block of negatively charged residues. The nature of HingeW suggests another benefit of the TBS library screening approach, in which directed evolution of the antagonist led to an improvement by reducing homodimerization. TBS has provided considerable utility in the exploration of novel sequence space by producing a protein sequence, which could not have been predicted without the use of this library screening approach.

TBS opens a new capability in semirational PPI design, where both affinity and activity are coselected. This offers significant potential to expand the TBS approach to both new libraries and targets where previous work may have produced potential antagonists, which were later found to lack functional activity. In principle, the approach can be fully expanded to any DNA binding protein that recognizes a discrete consensus sequence or even any dimeric system to which a DBD is appended. The method can be assumed to be generalizable since any DNA consensus sequence can be incorporated into the DHFR DNA sequence and can be transcriptionally blocked by coexpression of the relevant TFs. This will require the DHFR design process to be iterated and subsequent testing and optimization for each system; however, the central principle has been shown here to be valid. It also potentially permits the screening of exogenous molecules to allow concomitant profiling of both cell penetrant and functionally active inhibitors. Moreover, libraries with different design principles and expanded options harbor considerable additional promise in producing peptide hits across a broad range of targets in which pathogenic TFs are implicated. Library sizes of 10^6^–10^7^ are possible using standard techniques and readily available reagents, which may allow the exploitation of a broader range of peptide diversity and further optimization. Further TBS screening for a range of TF targets will produce both nongenetic tools and probes of disease pathways, but there is also considerable potential for a new generation of optimized functional antagonists and clinical leads.

## Materials and Methods

Proteins/peptides (sequences in Table S4) were produced using standard recombinant expression or solid-phase peptide synthesis methodologies and purified by various chromatography steps. DHFR activity was measured using a colorimetric assay kit (Sigma CD0340). Peptide affinity and antagonism were measured using established protocols for CD, ITC, and EMSA. A detailed description of the materials and methods utilized in this work is provided in the Supporting Information.

## Supporting Information Available

Detailed materials and methods, TRE-mDHFR sequence and purification, images of plates from assay optimization experiments, MTX inhibition of DHFR proteins, TBS screening sequencing data, figures detailing biophysical characterization, tables detailing TBS validation *E. coli* cell lines, *P* values from those validation experiments, biophysical characterization of peptides, and the peptide sequences (PDF)
