# Supplementary material for: An Approach to Derive Functional Peptide Inhibitors of Transcription Factor Activity
Source: JACS Au. 2022 Apr 6;2(4):996–1006. doi: 10.1021/jacsau.2c00105 (PMC9088798; doi:10.1021/jacsau.2c00105)
Supplement: Supplementary file 1 — au2c00105_si_001.pdf [file au2c00105_si_001.pdf]

Supporting Information:

An Approach to Derive Functional Peptide Inhibitors of Transcription Factor Activity.

Andrew Brennan[1], James T. Leech[2], Neil M. Kad[2], and Jody M. Mason[1*]

[1]Department of Biology & Biochemistry, University of Bath, Bath, BA2 7AY, UK.

[2]School of Biological Sciences, University of Kent, Canterbury, CT2 7NH, UK.

*Address correspondence to JMM (j.mason@bath.ac.uk)

Materials and Methods

Plasmid Constructs and Protein Production: The TRE-mDHFR (Figure S1) and WT-mDHFR DNA constructs were subcloned into pQE16 derivative plasmid pES300d; the cJun LZ and cJun bZIP DNA constructs were subcloned into pQE16 derivative plasmid pES230d; and the cFos LZ and A-FosW DNA constructs were subcloned into pET24a. The human cJun bZIP domain spans from Arg^{252} to Leu^{308} and the LZ domain spans from Ile^{277} to Leu^{308}. The human cFos bZIP domain spans from Glu^{137} to Leu^{193} and the LZ domain spans from Thr^{162} to Leu^{193}. A-FosW has the following sequence: LEQRAEELARENEELEKEAEELEQE**LDELQAEIEQLEERNYALRKEIEDLQKQLEKL** (FosW sequence in bold). All constructs are capped at the N-terminus with AS residues and at the C-terminus with GAP residues and are also 6xHis-tagged, other than the WT- or TRE-mDHFR constructs which are only 6xHis-tagged. A full list of sequences is provided in Figure S14.

Proteins were purified by subcloning their DNA sequences into either a pET21-His-SUMO plasmid (cJun bZIP, cFos bZIP) or a pET24a plasmid (HingeW, A-FosW, FosW) using NheI and AscI sites. An overnight culture of *E. coli* containing the relevant plasmid was used to inoculate LB media at a dilution factor of 1:1000. This culture was incubated with shaking (37°C, 200 rpm) until the OD_{600nm} reached 0.7. Protein over-expression was induced by the addition of IPTG (1 mM) before incubation with shaking (25°C, 200 rpm) overnight. Cells were then harvested from the culture by centrifugation. Cell pellets were resuspended in Histrap Binding Buffer (20 mM potassium phosphate, 500 mM NaCl, 40 mM imidazole, 5 mM DTT, pH 7.4), sonicated and loaded on a HisTrap HP 5 mL pre-loaded column. The column was washed with Binding Buffer before eluting protein samples on a Binding Buffer:Elution Buffer (20 mM potassium phosphate buffer, 500 mM NaCl, 400 mM imidazole, 5 mM DTT, pH 7.4) gradient. This methodology was also used to produce a ~80% pure sample of His-tagged ULP1 protease for use in the SUMO cleavage step. SUMO-tagged proteins were buffer exchanged into Standard Buffer (20 mM Tris.HCl, 2 mM DTT, pH 8.0). A 10:1 mixture of SUMO-tagged protein:ULP1 was incubated at 30°C for 16h. As the SUMO-tagged construct was N-terminally His-tagged on the SUMO, the cleavage reaction was diluted 1 in 5 in Binding Buffer and then passed through the HisTrap column to remove the cleaved SUMO tag and the His-tagged ULP1. The HisTrap flowthrough was finally purified to >98% purity by using RP-HPLC with a

Jupiter Proteo column (4-μm particle size, 90 Å pore size, 250 × 10 mm; Phenomenex) using a water:acetonitrile gradient (0.1% TFA). Peptides without a SUMO tag, were concentrated after Histrap elution and HPLC purified. Peptide purity and identity were verified by SDS-PAGE and electrospray ionisation mass spectrometry.

DHFR Activity Assay: A colorimetric assay kit (Sigma CD0340) was used to measure the activity of purified DHFR enzymes. WT- or TRE-mDHFR (100 nM in reaction) and NADPH (60 μM in reaction) were mixed in assay buffer only, or with DHFR inhibitors TMP or Mtx (1 μM in reaction). Reactions were initiated by the addition of DHF (50 μM in reaction plus a blank reaction with no DHF) and the absorbance at 340 nm of samples was measured using a Varian Cary 50 UV-Vis spectrophotometer. The specific activity was calculated using the following equation. $Specific\ activity = \frac{(\Delta OD/min_{sample} - \Delta OD/min_{blank})}{12.3 \times mg\ protein}$

Library Construction and TBS Assay: Library inserts were produced using PCR fill-in reactions from synthesised primers (Sigma) with degenerate codons at the desired positions to produce the correct residue options. The library was subcloned using SacI and AscI sites into the pET24a plasmid containing A-FosW. The primers used were cJun-Hinge-Lib-F: 5'-GAAGAGCTCSWGSWGSWGSWGSWTSWGCTGSWGGMASWGATTGAACAGCTGGAAGAACGCAACTATGCC-3' and cJun-Hinge-Lib_R: 5'-TGAGGCGCGCCCAGTTTCTCCAGCTGTTTCTGGAGGTCTTCGATCTCTTTGCGCAAGGCATAGTTGCGTTC-3'. The library DNA was transformed into NEB 10-beta electrocompetent *E. coli* cells. The following equation was utilised to determine library coverage by the number of single colonies: $E = 100 \times (1 - \frac{1}{n})^m$ where E is the percentage of the library missing, m is the number of colonies collected and n is the library size. This showed that from 2155000 library colonies collected, 99.9% of the Hinge library was covered. Library DNA quality was assessed by sequencing both the DNA pool and a number of single colonies to show degenerate codons in the correct positions in the pool and to show a diversity of library members from single colonies. The pool of library DNA was transformed into BL21 Gold cells already containing pES300d-TRE-mDHFR and pES230d-cJun bZIP.

Selective pressure is applied by growing the bacteria in M9 minimal media with TMP (2-4 μM) alongside ampicillin, kanamycin and chloramphenicol to maintain the required plasmids, and IPTG (1 mM) to induce protein expression. The library transformants were first plated out onto selective agar plates (2 μM TMP) and grown at 37°C for 72-96h. Optimisation experiments (Figure S3) indicate that 4 μM TMP is optimum for selection however a lower stringency is used initially before selection is increased in later steps. Colonies from this first round of selection were pooled and serially grown in liquid culture at starting OD_{600} of 0.05 and grown at 37°C with shaking at 200 rpm until the OD_{600} reached 0.6. TMP concentration was 2 μM in the first liquid culture passage before it was increased to the optimum 4 μM in subsequent passages. Bacteria containing the most effective functional antagonists were expected to produce higher levels of TRE-mDHFR which provides a growth advantage, and these will dominate the culture. At each passage step, a sample of the culture was plated on LB agar (supplemented with antibiotics to maintain plasmids) to select and sequence individual colonies, and a DNA pool was also sequenced. This allows the occurrence of library members to be monitored as winner sequences are selected for. Assay Validation experiments utilised a modified assay methodology whereby overnight cultures from glycerol stocks of the Control Strains (Figure S6) were diluted to OD_{600}=0.5 and 50 μL was plated on a selective M9 minimal medium agar plate.

Circular Dichroism (CD): An Applied Photophysics Chirascan was used for CD measurements, with a 200 μL sample in a 1 mm path length CD cell. Protein/DNA samples were suspended in 150 mM potassium phosphate,150 mM potassium fluoride and 5 mM TCEP at pH 7.4 and were equilibrated for 30 minutes before measurement. For full spectra, three scans between 190 and 260 nm (265-320 nm for DNA binding experiments) were collected with a bandwidth of 1 nm and data sampled at a rate of 0.5 s^{-1}. These scans were averaged, a blank was subtracted, and the values were converted to molar residue ellipticity (MRE). Thermal denaturation experiments were performed by measuring the ellipticity at 222 nm over a 1 to 90°C gradient at 1°C increments. Post-melt scans at 20°C confirmed the transitions were reversible as they overlaid within 10% of the pre-melt scan. The resulting thermal denaturation curves were converted to MRE and fitted to a two-state model, derived *via* modification of the Gibbs–Helmholtz equation to determine the melting temperature (T_m) (*44*).

Isothermal Titration Calorimetry (ITC): Peptides were studied in an ITC buffer consisting of 10 mM potassium phosphate, 150 mM potassium fluoride and 5 mM TCEP at pH 7.4. Using a MicroCal VP-ITC instrument (Malvern), 10 μL injections of antagonist peptide (HingeW or A-FosW) at 10 μM were injected into the cell containing cJun at 1 μM. MicroCal Origin software was used to record and analyse the heat change upon addition. Control experiments involved the injection of the antagonist peptide sample into the cell containing ITC buffer alone to determine the heat of dilution which was subtracted. The resulting binding data were fit to a one site binding model to extract the enthalpy change of binding (ΔH) and the equilibrium binding constant (K_D), from which the free energy change of binding (ΔG) and the entropy change of binding (ΔS) was calculated (*41*). Thermodynamic parameters are presented as an average of two independent experiments with errors given as one standard deviation.

Electrophoretic Mobility Shift Assay (EMSA): The following double-stranded oligonucleotide sequences were used, TRE: 5'-GTCAGTCAGTGACTCAATCGGTCA, control non-TRE: 5'-CCTGCGTAGTTCCATAAGGATAGC (Sigma). Complementary single strands of DNA were purchased (Sigma) and mixed at a 1:1 ratio, then heated to 95°C for 20 minutes before cooling slowly to room temperature to form DNA duplexes. Protein/DNA samples for electrophoresis were incubated at 4°C for 30 minutes in binding buffer (150 mM KCl, 1 mM dithiothreitol, 1 mM EDTA, 10 mM Tris, 10 mM $MgCl_2$, pH 8) before running on a 1.3% agarose gel in 0.5xTBE buffer (supplemented with 10 mM $MgCl_2$). SYBR® Green stain was included in the gel and running buffer to stain for DNA which was imaged on a transilluminator before SYPRO® Ruby was added and incubated for 3 h to stain for protein. The gel was destained in a 10% methanol, 7% acetic acid solution for 1 h before imaging on a transilluminator.

A S V R P L N C I V A V S Q N M G

GCT AGC GTT CGA CCA TTG AAC TGC ATC GTC GCC GTG AGT CAG AAT ATG GGG

I G K N G D L P W P P L R N E S K

ATT GGC AAG AAC GGA GAC CTA CCC TGG CCT CCG CTC AGG AAT GAG TCA AAG

Y F Q R M T Q T D S V E S K Q N L

TAC TTC CAA AGA ATG ACT CAG ACT GAC TCA GTT GAG TCA AAA CAG AAT CTG

V I M G R K T W F S I P E S N R P

GTG ATT ATG GGT AGG AAA ACC TGG TTC TCC ATT CCT GAG TCA AAT CGA CCT

L K D R I N I V L S Q E L K E P P

TTA AAG GAC AGA ATT AAT ATA GTT CTG AGT CAA GAA CTC AAA GAA CCA CCA

R G A H F L A K S L D D A L R L I

CGA GGA GCT CAT TTT CTT GCC AAA AGT TTG GAT GAT GCC TTA AGA CTT ATT

E S P E L A S K V D S V W I V G G

GAG TCA CCG GAA TTG GCG AGC AAA GTT GAC TCA GTT TGG ATC GTC GGA GGC

S S V Y Q E A M T Q P G H L R L F

AGT TCT GTT TAC CAG GAA GCC ATG ACT CAA CCA GGC CAC CTT AGA CTC TTT

V T Q I M Q E F E S D T F F P E I

GTG ACT CAG ATC ATG CAG GAA TTT GAG TCA GAC ACG TTT TTC CCA GAA ATT

D S G K Y K L L P E S P G V L S Q

GAC TCA GGG AAA TAT AAA CTT CTC CCT GAG TCA CCA GGC GTC CTG AGT CAG

V Q E E K G I K Y K F E V Y E K K

GTC CAG GAG GAA AAA GGC ATC AAG TAT AAG TTT GAA GTC TAC GAG AAG AAA

D * A *

GAC TAA GCT TAA

Figure S1. Protein and DNA sequence of TRE-mDHFR showing the introduction of 15 TRE sites into the gene. Amino acids and DNA bases mutated from the WT murine protein are shown in red, and the TRE DNA sites these mutations have added are emboldened and underlined. Shown in green are the NheI and HindIII sites used for subcloning the gene into the pES300d vector.

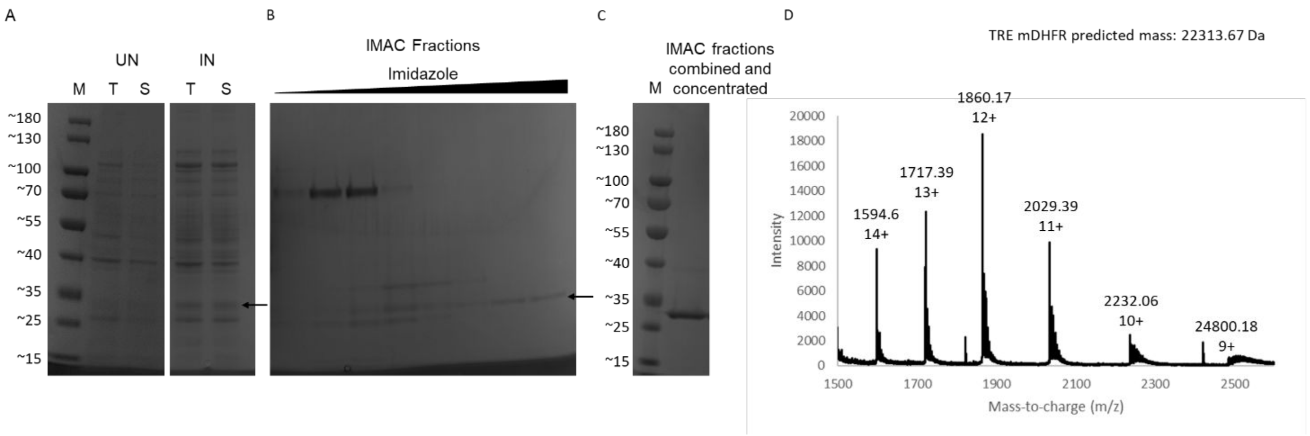

Figure S2. TRE-mDHFR is expressed in the soluble fraction and can be purified for further study. (A) SDS-PAGE analysis of *E. coli* cell lysate from cells before and after the induction of TRE-mDHFR plasmid expression with IPTG (1 mM, 18 hours, 30°C). A total (T) sample is taken directly after lysis and a soluble (S) sample is taken after the lysate is centrifuged. This shows the appearance of a protein band in the induced samples with equal band intensity in the T and S fractions, indicating the protein is folded and soluble. TRE-mDHFR can be bound to an immobilised metal affinity chromatography column due to its 6xHis-tag and subsequently eluted by an imidazole gradient (B) to give pure protein as determined by (C) SDS-PAGE of the combined and concentrated fractions shown to contain the induced protein band. TRE-mDHFR did not migrate through the polyacrylamide gel as predicted by the protein marker lane (M), running at an apparently higher molecular weight but its identity was confirmed by (D) electrospray ionisation mass spectrometry.

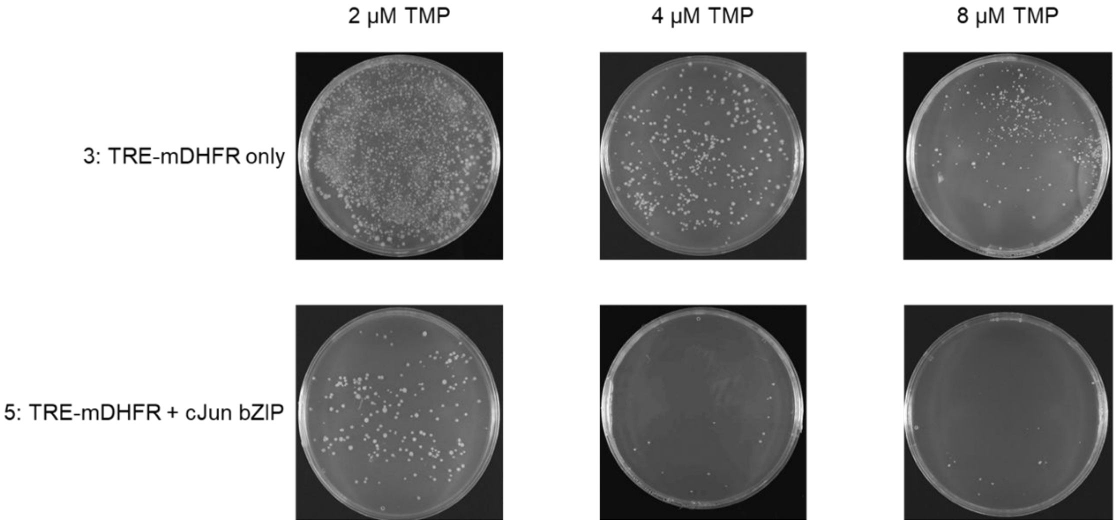

Figure S3. Optimisation of TMP concentration required to produce selectivity between *E. coli* expressing TRE-mDHFR and *E. coli* with TRE-mDHFR expression transcriptionally-blocked by cJun bZIP. Controlled numbers of *E. coli* cells expressing the indicated proteins were plated on selective media at varying TMP concentration. 4 μM TMP produces the optimum differential in colony numbers between the TRE-mDHFR only and TRE-mDHFR + cJun bZIP plates.

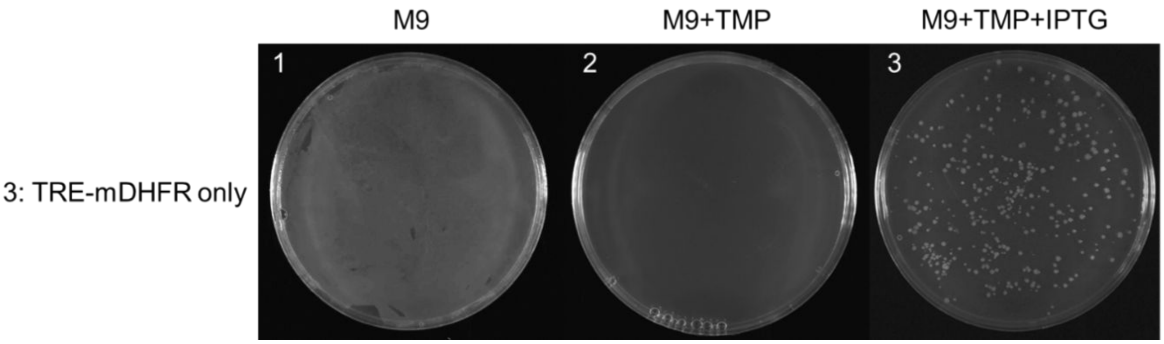

Figure S4. Bacterial DHFR can be inhibited by TMP and have its activity replaced by the induction of TRE-mDHFR expression. *E. coli* cells containing the plasmid for TRE-mDHFR only grow differentially on different agar media, following the design principles of the TBS assay. In M9 agar (1), a lawn of colonies is produced as the bacteria grow freely; upon addition of TMP (2) to the media the bacterial DHFR is inhibited and cells cannot grow; and further addition of IPTG (3) leads to expression of the TRE-mDHFR which restores cell survival to a degree.

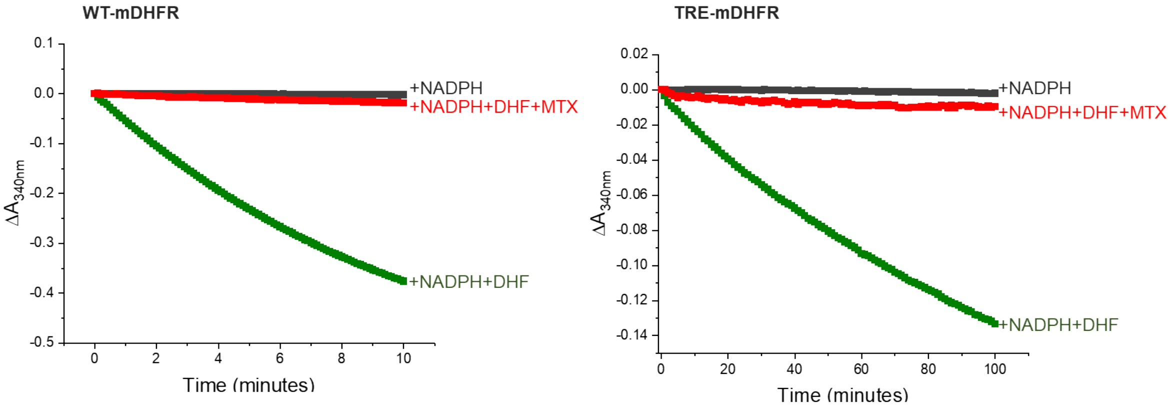

Figure S5. Both WT- and TRE-mDHFR are inhibited by the broad DHFR inhibitor MTX. The change in absorbance at 340 nm was measured to determine the rate of NADPH turnover by WT-mDHFR and TRE-mDHFR, with and without the substrate DHF. Also shown are the reactions repeated in the presence of MTX which shows clear inhibition of the reaction, as expected and indicative of DHFR activity.

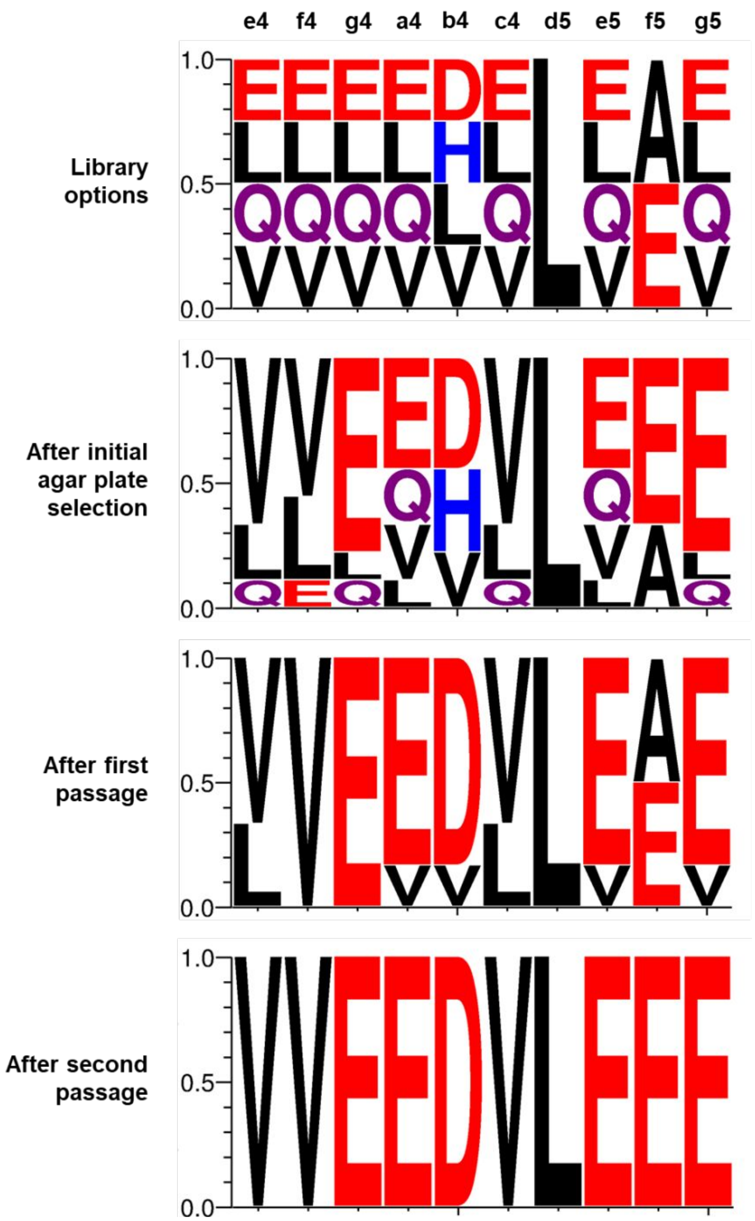

Figure S6. TBS selection pressure shifts the representation of library members in the DNA pool sequencing towards the selection of HingeW as an assay winning sequence. Sequence logos showing the relative abundances of the amino acids on the initial selection plate (nine colonies sequenced), the first passage (six colonies sequenced) and the final winning sequence (HingeW; only sequence present in the DNA pool and in five colonies). Small sample sizes may not be fully representative but simply indicate sequences which are present, further the DNA pool sequencing supports these trends.

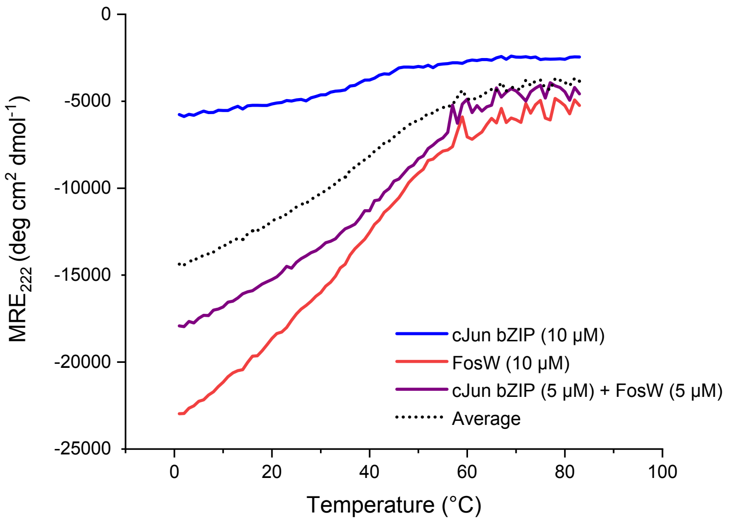

Figure S7. CD thermal denaturation profiles showing the interaction of FosW with the cJun bZIP. The thermal denaturation profile of the FosW/cJun bZIP heterodimer is shifted from the average of the two component peptide curves, highlighting an increased helicity and T_m value (54°C for the heterodimer), indicative of a binding interaction.

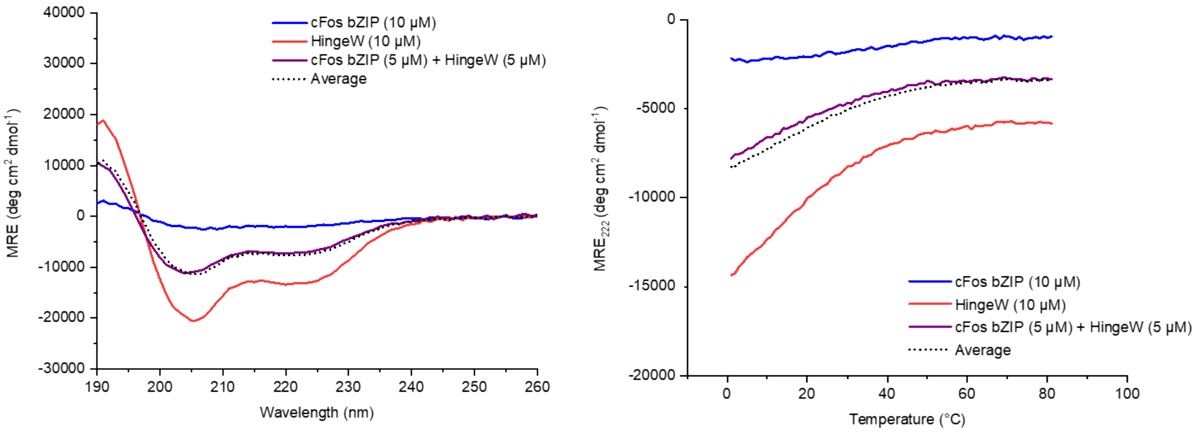

Figure S8. TBS winner peptide HingeW does not interact with cFos. CD spectra and thermal denaturation curves showing no interaction between HingeW and cFos as the measured heterodimer spectrum/thermal denaturation curve overlays with the average of the individual component spectra.

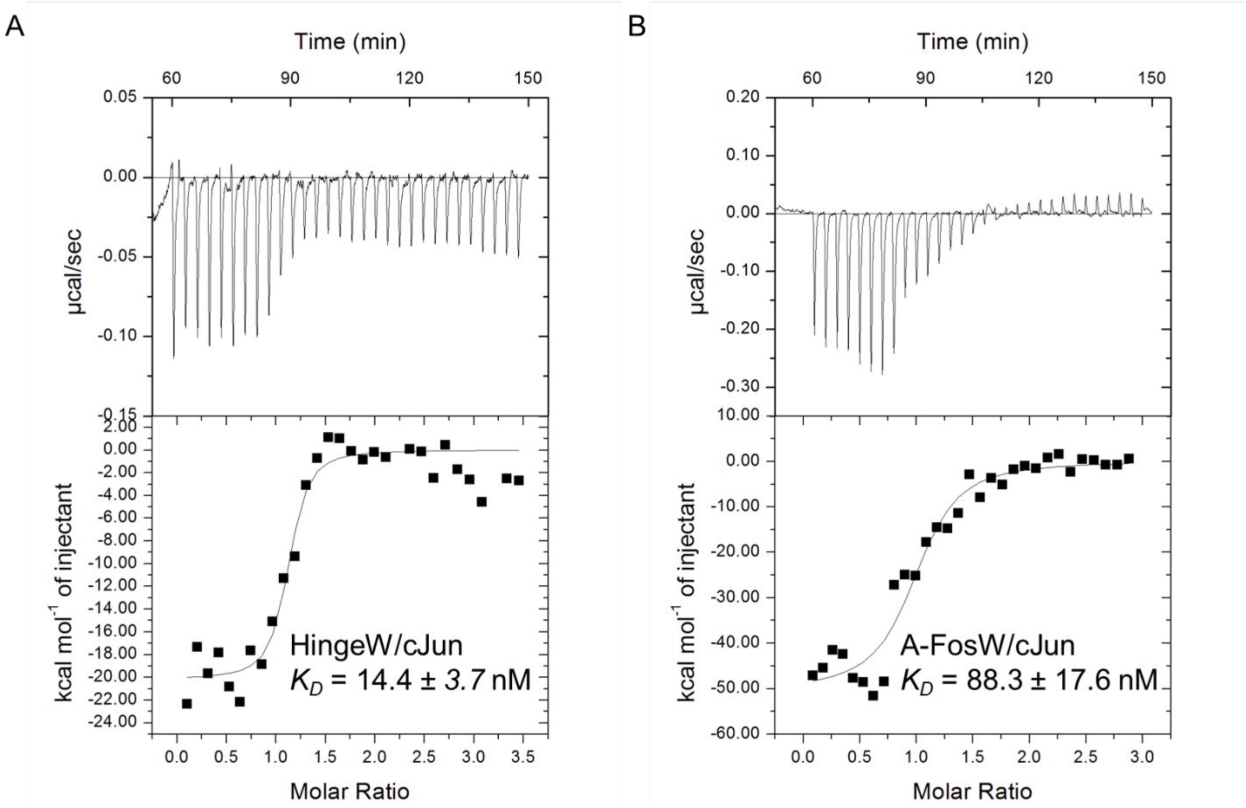

Figure S9. Isothermal titration calorimetry data demonstrate a six-fold higher affinity for HingeW/cJun relative to A-FosW/cJun. ITC analysis profiles for cJun binding to (A) HingeW and (B) A-FosW show the raw power compensation plot throughout the titration in the upper graph and the integrated data points and single site model fit (MicroCal ORIGIN software) in the lower graph. Errors shown as one standard deviation.

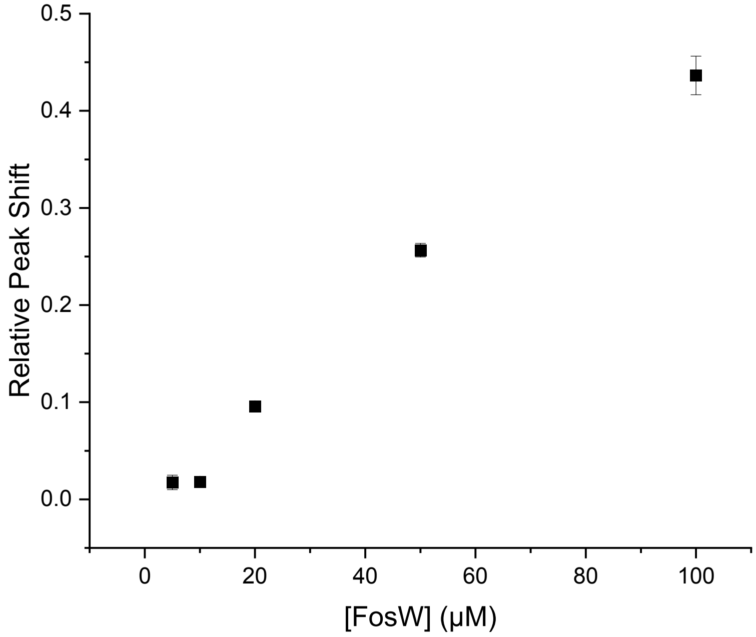

Figure S10. CD antagonism data showing the shift in the DNA spectrum upon addition of FosW to the cJun-bound DNA. The relative shift from the cJun-bound TRE DNA peak to the free TRE DNA peak is monitored at 281 nm as FosW is sequentially added. Data averaged from three independent experiments.

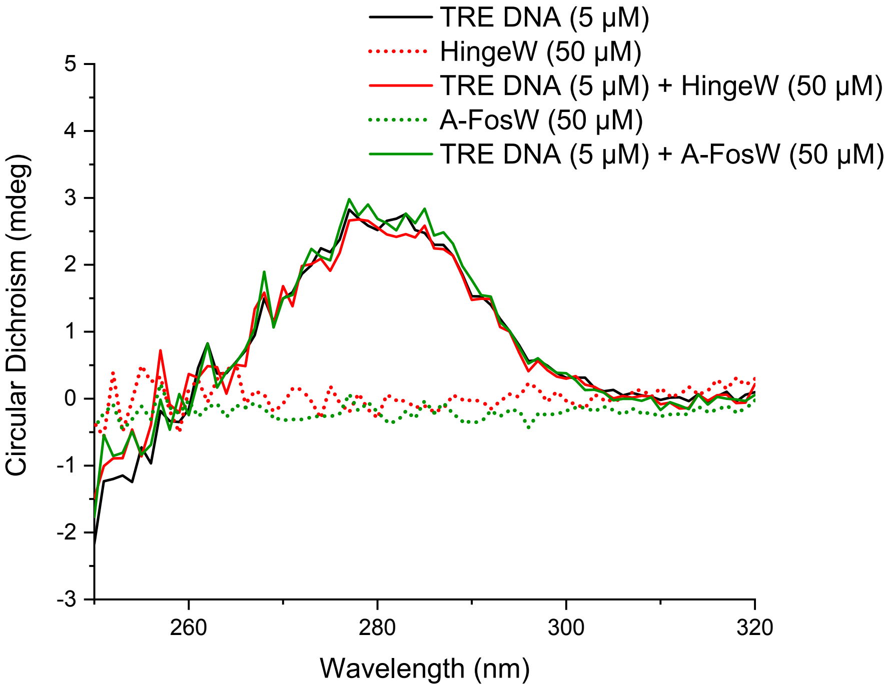

Figure S11. HingeW and A-FosW do not interact with TRE DNA. CD spectra demonstrates no interaction between either HingeW or A-FosW with TRE DNA. Proteins do not absorb in this wavelength range, so the CD signal observed occurs due to the DNA structure, which is not perturbed upon addition of either protein.

	pES300d	pES230d	pET24a
1	WT-mDHFR	Scrambled (cJun bZIP)	Scrambled (A-FosW)
2	WT-mDHFR	cJun bZIP	Scrambled (A-FosW)
3	TRE-mDHFR	Scrambled (cJun bZIP)	Scrambled (A-FosW)
4	TRE-mDHFR	cJun LZ	Scrambled (A-FosW)
5	TRE-mDHFR	cJun bZIP	Scrambled (A-FosW)
6	TRE-mDHFR	cJun bZIP	cFos LZ
7	TRE-mDHFR	cJun bZIP	FosW
8	TRE-mDHFR	cJun bZIP	A-FosW
9	TRE-mDHFR	cJun bZIP	HingeW

Table S1. TBS assay validation *E. coli* control strains showing inserts expressed from each plasmid. Strains for growth experiments are plasmid matched to ensure equal antibiotic pressure, using scrambled protein sequences in unrequired plasmids.

	2	3	4	5	6	7	8	9
1	4.3E-02	3.2E-06	3.2E-06	3.1E-06	4.6E-05	4.6E-05	4.6E-05	4.6E-05
2		9.3E-06	9.3E-06	9.1E-06	1.1E-04	1.1E-04	1.1E-04	1.1E-04
3			1.4E-01	8.7E-05	4.9E-04	5.8E-04	2.2E-02	3.8E-02
4				2.0E-06	1.6E-05	2.0E-05	7.0E-03	2.4E-02
5					8.1E-01	2.2E-01	1.4E-06	7.2E-07
6						1.1E-02	1.2E-06	3.6E-07
7							1.0E-06	2.6E-07
8								9.5E-03

Table S2. Table of *P* values showing statistical differences between the colony counts of the tested *E. coli* control strains. Dark red boxes indicate $P \leq 0.0001$, red boxes indicate $P \leq 0.001$, orange indicates $P \leq 0.01$, yellow indicates $P \leq 0.05$, white indicates $P > 0.05$.

	Fraction helicity at 20°C (CD)	T_m (°C) (CD)	ΔT_m from component average (CD)	K_D (nM) (ITC)	N (ITC)	ΔG (KJ mol^{-1}) (ITC)	ΔH (KJ mol^{-1}) (ITC)	$T\Delta S$ (KJ mol^{-1}) (ITC)
**cJun**	14.0%	39.8 ± 1.9	-	-	-	-	-	-
**A-FosW**	40.1%	45.1 ± 0.9	-	-	-	-	-	-
**A-FosW/cJun**	38.7%	69.9 ± 0.7	27.5	88.3 ± 17.6	1.04 ± 0.08	-40.0± 0.5	-152.6 ± 4.1	-112.6 ± 4.1
**HingeW**	27.2%	-	-	-	-	-	-	-
**HingeW/cJun**	34.7%	71.2 ± 1.4	~40	14.4 ± 3.7	1.05 ± 0.05	-46.2 ± 0.6	-85.4 ± 4.5	-39.2 ± 4.5

Table S3. Thermodynamic parameters for the interactions between cJun and either the rationally designed A-FosW template or the TBS library derived HingeW. Errors shown as one standard deviation.

Peptide	Sequence
cJun LZ	MASIARLEEKVKTLKAQNYELASTANMLREQVAQLGAPHHHHHH
cJun bZIP	RIKAERKRMRNRIAASKCRKRKLERIARLEEKVKTLKAQNYELASTANML REQVAQLGAP
cFos LZ	MASTDTLQAETDQLEDEKYALQTEIANLLKEKEKLGAPHHHHHH
cFos bZIP	EEKRRIRRERNKMAAAKCRNRRRELTDTLQAETDQLEDEKYALQTEIANL LKEKEKLGAP
FosW	MASLDELQAEIEQLEERNYALRKEIEDLQKQLEKLGAPHHHHHH
A-FosW	MASLEQRAEELARENEELEKEAEELEQELDELQAEIEQLEERNYALRKEIE DLQKQLEKLGAPHHHHHH
HingeW	MASLEQRAEELARENEELEKEAEELVVEEDVLEEEIEQLEERNYALRKEIE DLQKQLEKLGAPHHHHHH

Table S4. Peptide sequences.
